# Perception of rotation, path, and heading in circular trajectories

**DOI:** 10.1007/s00221-016-4638-0

**Published:** 2016-04-07

**Authors:** Suzanne A. E. Nooij, Alessandro Nesti, Heinrich H. Bülthoff, Paolo Pretto

**Affiliations:** Department of Human Perception, Cognition and Action, Max Planck Institute for Biological Cybernetics, Tübingen, Germany

**Keywords:** Motion perception, Heading, Eccentric rotation, Translation, Psychophysics, Vestibular, Multisensory integration

## Abstract

When in darkness, humans can perceive the direction and magnitude of rotations and of linear translations in the horizontal plane. The current paper addresses the integrated perception of combined translational and rotational motion, as it occurs when moving along a curved trajectory. We questioned whether the perceived motion through the environment follows the predictions of a self-motion perception model (e.g., Merfeld et al. in J Vestib Res 3:141–161, 1993; Newman in A multisensory observer model for human spatial orientation perception, 2009), which assume linear addition of rotational and translational components. For curved motion in darkness, such models predict a non-veridical motion percept, consisting of an underestimation of the perceived rotation, a distortion of the perceived travelled path, and a bias in the perceived heading (i.e., the perceived instantaneous direction of motion with respect to the body). These model predictions were evaluated in two experiments. In Experiment 1, seven participants were moved along a circular trajectory in darkness while facing the motion direction. They indicated perceived yaw rotation using an online tracking task, and perceived travelled path by drawings. In Experiment 2, the heading was systematically varied, and six participants indicated, in a 2-alternative forced-choice task, whether they perceived facing inward or outward of the circular path. Overall, we found no evidence for the heading bias predicted by the model. This suggests that the sum of the perceived rotational and translational components alone cannot adequately explain the overall perceived motion through the environment. Possibly, knowledge about motion dynamics and familiar stimuli combinations may play an important additional role in shaping the percept.

## Introduction

The vestibular system, with its semicircular canals (SCCs) and the otoliths, plays a major role in spatial orientation, navigation, and the perception of self-motion (see Angelaki and Cullen 2008 for a review). By studying motion perception during simple motions in the horizontal plane—like single translations or rotations—several perceptual mechanisms have been identified that describe how the vestibular signals are transformed into an overall percept of one’s motion through the environment. These will be laid out below. In the current study, we questioned whether these mechanisms, which are formalized in a model of self-motion perception, are also sufficient to understand the perception of more complex, curved motion paths, where rotational and translational components are combined. To that end, we compared model predictions with experimentally obtained measures of the perceived self-motion through the environment.

The SCCs respond to angular accelerations of the head, but, due to their dynamics, act as velocity transducers in the frequency range of natural head movements (Guedry 1974). That means that their response is proportional to the rotational velocity of the head. The perceived angular displacement is then computed by the central nervous system (CNS) by integrating the perceived velocity over time, as shown for earth vertical rotations (e.g., Israël et al. 1995; Mergner et al. 1996). The otoliths are sensitive to the net linear acceleration of the head. Their signal is ambiguous, in the sense that they respond to both gravity (i.e., head tilt) and inertial motion (i.e., translation). By integrating both SCC and otolith signals, the CNS can resolve this ambiguity and obtain an estimate of both the gravitational and inertial acceleration (e.g., Merfeld et al. 1999, 2001; Zupan and Merfeld 2003; Angelaki et al. 2004; Yakusheva et al. 2007). The perceived inertial acceleration is subsequently transformed in a translation estimate. Indeed, when being moved along a linear track in darkness, humans can reliably detect their heading [i.e., the direction of motion with respect to their body (Telford et al. 1995; Butler et al. 2010; De Winkel et al. 2010; MacNeilage et al. 2010a)] and they can derive linear velocity and travelled path by integrating the perceived linear acceleration over time (Israël et al. 1989, 1993; Mittelstaedt and Mittelstaedt 2001; Yong et al. 2007; Seidman 2008).

Both the SCC and otolith systems are optimized for natural head movements, and perception becomes inaccurate at lower frequencies. For the SCCs, this is best shown during constant velocity rotation in darkness, where the perceived rotational velocity decays over time (e.g., Guedry 1974; Mittelstaedt and Mittelstaedt 1996). Similarly, perceived linear velocity has also been shown to decay over time when the linear velocity is constant (Seidman 2008). In addition, prolonged linear acceleration in darkness is known to induce a feeling of tilt (i.e., somatogravic illusion, Guedry 1974; Merfeld et al. 2001; Clement et al. 2002; Correia Grácio et al. 2013). This occurs because at low-frequency motions the response of the SCCs is attenuated, which impedes the separation of tilt and translation. As a solution, the CNS interprets the net otolith signal (which is tilted with respect to the head) as being the gravitational vertical, since this is the main omnipresent constant acceleration humans are exposed to. Because tilt and translation are directly connected (i.e., the inertial and gravitational acceleration both add up to the total otolith signal), the illusory tilt results in an underestimation of the perceived translation (Glasauer 1995; De Graaf et al. 1996; Seidman et al. 1998; Merfeld et al. 2005a).

In sum, motion perception in darkness can be characterized by four main mechanisms: (1) the integration of SCC and otolith signals to resolve tilt and translation (tilt-translation resolution); (2) the somatogravic effect; (3) the decay in the perceived rotational and translational velocity during constant velocity motion; and (4) the integration of perceived velocity over time to obtain a translation estimate. In the current paper, we investigated whether these mechanisms are also sufficient to understand motion perception during motions where the rotational and translational components are combined in the horizontal plane, as it occurs when moving through a curve. The off-center yaw rotation induces tangential and centripetal linear accelerations that act on the body and have to be integrated with the rotational velocity to form an overall percept of the motion through the environment. Is the overall perceived motion the sum of its perceived rotational and translational components?

Earlier work on this topic has been done by Ivanenko et al. (1997b), who argued that this does not seem to be the case. They exposed participants to different combinations of translational and rotational motion cues in the horizontal plane in darkness and had them draw the perceived motion path afterward. Trajectories included 180° curved motions, where the head and body orientation was always toward the inner or outer side of the curve, toward the direction of motion, or in a fixed orientation with respect to an external landmark. The overall percept was not veridical and appeared to be dominated by the perceived rotation. Participants only drew veridical motion paths when the stimulus pattern was familiar (like going through a curve while facing the direction of motion). From these results, the authors concluded that the overall motion percept was not equal to the sum of the separate translational and rotational motion components.

**Fig. 1:**
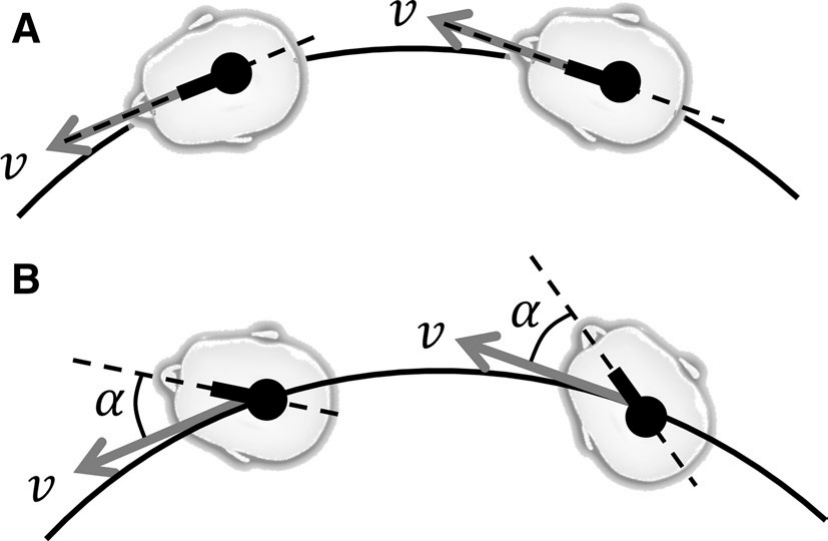
**a** Example of the “straight-ahead” heading, where one is aligned with the motion direction. **b** The heading (*α*) is the angle between the instantaneous linear velocity vector $v$ and the *body midline*

Here, we built upon these earlier results and investigated whether motion perception in curved trajectories can be understood by the four main perceptual principles presented above. As pointed out earlier, accuracy is compromised for motions at the lower frequencies: Decay in the perceived translation or rotation, combined with the possible illusion of tilt, makes the overall percept deviate from the actual motion profile. As these perceptual principles are formalized in mathematical models of self-motion perception (*perception model* for short), we took the predictions of a current motion perception model as a starting point and compared these to several motion perception measures that were experimentally obtained. Specifically, we investigated perceived yaw rotation (i.e., angular displacement), perceived motion path, and perceived heading during circular trajectories. Here, heading is expressed in an egocentric reference frame. It is defined as the instantaneous direction of linear velocity—always directed tangentially to the curve—with respect to the body’s instantaneous “straight-ahead” direction (i.e., the body midline, Stone and Perrone 1997; Li et al. 2006). The heading thus reflects one’s orientation with respect to the motion path. As shown in Fig. 1a, a 0° or “straight-ahead” heading indicates that one is exactly facing the motion direction, whereas a nonzero heading indicates that one is rotated with respect to the motion path, facing to the outer side (as in Fig. 1b) or the inner side of the curve.

### Perception model and predictions

Predictions of self-motion percepts were computed using the model of Newman (2009), which is an extended version of the model of Merfeld et al. (1993). This latter model was originally developed to model vestibular driven ocular reflexes, but it has also been applied to model self-motion perception in both simple and complex motion paradigms (Merfeld et al. 2005a, b; Vingerhoets et al. 2006, 2007; Clark et al. 2015). It describes sensory information processing during self-motion and accounts for the dynamics of the sensors, the tilt-translation resolution, and the somatogravic illusion. Based on the vestibular input signals, it provides estimates of perceived tilt, linear acceleration, and rotational velocity. Recently, the model has been extended to include outputs for heading, linear velocity, and displacement, which can be combined to provide an estimate of one’s overall motion through the environment. Moreover, it now also includes the integration between visual and vestibular signals (Newman 2009; Newman et al. 2012). A more detailed description of the extended model can be found in Appendix 1.

**Fig. 2:**
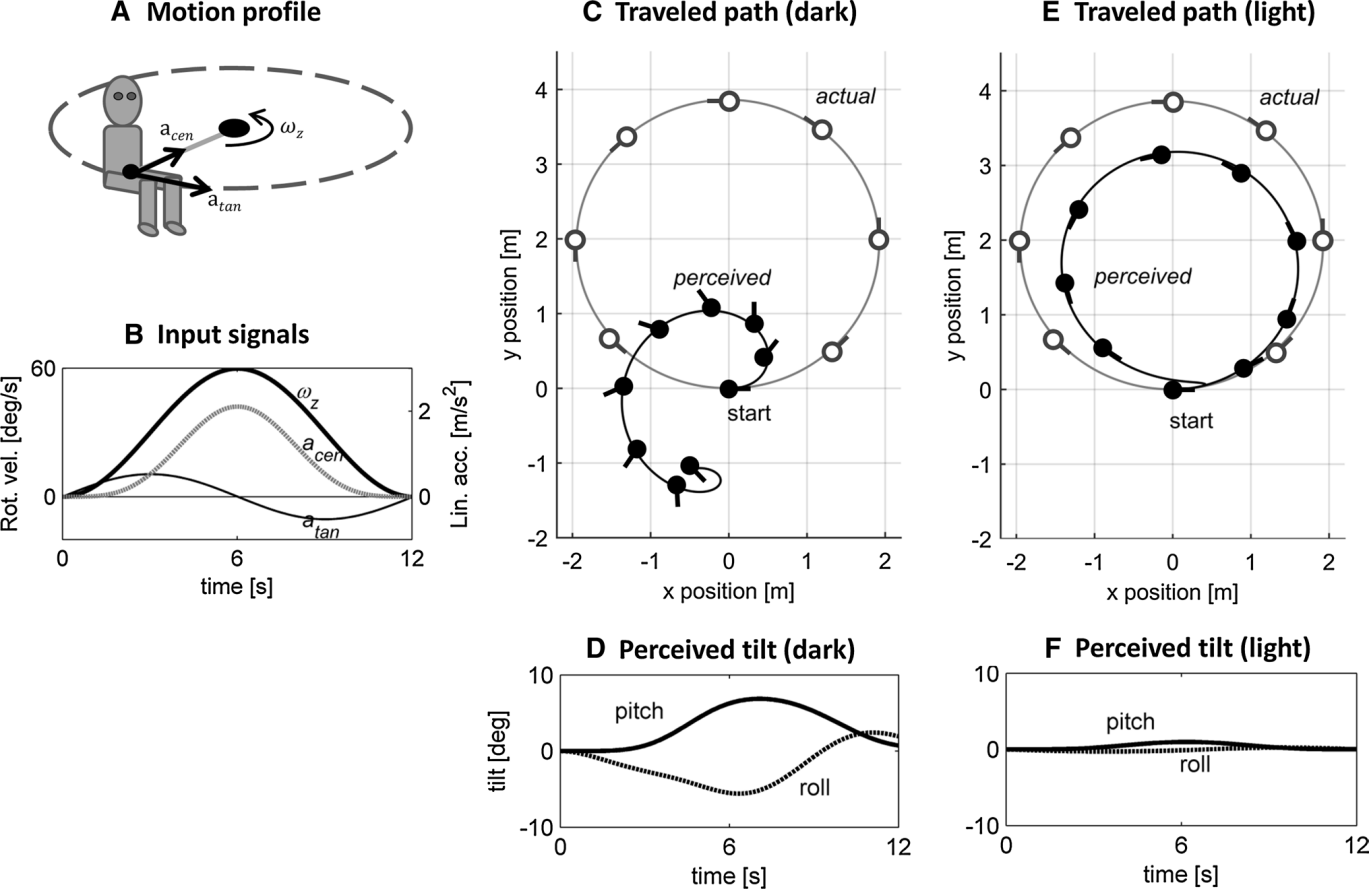
Predicted perceived motion for a 360° off-center circular motion in the horizontal plane (radius = 1.93 m) while being upright. **a** Schematic of the motion profile. **b** Input signals acting on the body, where $\omega_{z}$ = yaw velocity, $a_{\tan }$ = tangential acceleration, equal to $\omega_{z}^{2} \cdot R$, and $a_{\text{cen}}$ = centripetal acceleration, equal to $\dot{\omega }_{z} \cdot R$. **c**–**f** Model predictions. **c**, **e** A *topview* of the actual (*gray*, *open symbols*) and perceived traveled path (*black*, *filled symbols*) in darkness and light, respectively. The *dots* represent the head, with the outcoming *line* indicating the direction of the body midline (“the nose”). **d**, **f** Perceived tilt in darkness and light, respectively

Here, we show what the model predicts for the perception of a simple, circular motion. In this example, the body is moved along a circular trajectory in the horizontal plane—like a car going around a roundabout—while being erect and always facing the motion direction (Fig. 2a). The head is assumed to be fixed with respect to the body. The exact motion cues acting on the body, forming the input to the model, are depicted in Fig. 2b. The rotational velocity, in this case following a bell-shaped profile, is directed along the vertical body axis. This causes a tangential acceleration (associated with the angular acceleration) directed along the forward body axis and a centripetal acceleration (associated with the angular velocity) directed along the lateral body axis. Figure 2c shows the actual, circular motion trajectory through the environment as seen from the topview, together with the predicted perceived traveled path, in darkness. The orientation of the head is indicated by the circular symbols with the outcoming line indicating the direction of the body midline (“the nose,” see also Fig. 1). By comparing the actual motion (gray line, open symbols) with the predicted perceived motion (black line, filled symbols), it can be seen that the latter is far from veridical. Firstly, the overall rotation of the head in space, assessed by comparing the orientation of the head at the start and end of the trajectory, is smaller than 360°. This illustrates the fact that human perception of rotations is attenuated at low frequencies. Secondly, the perceived travelled path is considerably distorted: It has an ear-like shape instead of a circle, with considerable sideways motion present at the start of the trajectory. Finally, the perceived heading is no longer “straight ahead,” but biased toward the direction of rotation. That is, during a circle to the left (i.e., counterclockwise), the perceived instantaneous direction of motion is left of the body midline. In other words, this motion would be perceived as facing to the outside of the perceived curve.^1^

The distorted heading percept is mainly the result of the somatogravic effect: The perceived gravity vector is drawn toward the net otolith input leading to an illusory percept of pitch and roll (Fig. 2d). This perceived tilt directly affects the perceived inertial acceleration and thus also the motion path. Suppressing the tilt perception, for instance by providing visual information, indeed leads to a more veridical motion percept (Fig. 2e, f). Here, the visual motion information that was used as an input to the model consisted of one’s actual velocity and orientation with respect to gravity. Note that the perceived travelled path is not yet equal to the physical motion because of leakage in the rotational and translational components. This can be attenuated by providing visual information about absolute position. A second way to show the effect of the tilt percept on the overall percept is to disable the parts of the model that are responsible for the somatogravic effect, as is done in Appendix 2. This also leads to a more veridical motion percept.

### Study overview

In this study, two experiments were performed to test the model predictions. In Experiment 1, participants were repeatedly exposed to the circular trajectory depicted in Fig. 2a in darkness and they indicated both perceived angular displacement (by means of an online tracking task) and perceived traveled path (by means of a drawing after stimulus completion), similar to the measures taken by Ivanenko et al. (1997b). This provided a general picture of the perceived motion in terms of translation and rotation. In Experiment 2, we specifically investigated the perceived heading. During a similar circular motion trajectory, the participants’ heading was systematically varied between trials and, using a 2-alternative forced-choice task, participants indicated whether they felt being faced inward or outward at one specific moment halfway through the trajectory. In this way, we identified the physical heading that was perceived as the “straight ahead” (i.e., nose aligned with the motion direction, see Fig. 1). As mentioned above, when the physical orientation is tangential to the curve (i.e., “straight ahead”), the model predicts that a perceived outward orientation develops over the course of the trajectory (Fig. 2). Thus, in order to *perceive* one’s orientation as tangential to the curve, the physical orientation should be inwards. The difference between the physical and perceived orientation will be referred to as *heading bias*. The second experiment was also repeated with a congruent visual stimulus, to investigate whether a possible heading bias in darkness would indeed disappear when visual information was present. This would also be in line with the work of Stone and Perrone (1997), who showed that humans are able to perceive the heading on curved paths quite accurately, based on visual information alone.

## Experiment 1: angular displacement and motion path

The goal of Experiment 1 was to identify the perceived motion path and the perceived yaw rotation in space during circular motions. This experiment was performed in darkness only.

### Methods

In total, seven healthy volunteers (four males and three females, aged 21–34) participated. Prior to the study, they were informed about the general study objectives and methods. They all gave written informed consent and confirmed that they were free from any known vestibular, neurological, cardiac, or spinal illnesses. The study protocol was approved by the Medical Ethical Committee of the Karl Eberhard University of Tübingen, and the study was conducted in accordance with the Declaration of Helsinki.

**Fig. 3:**
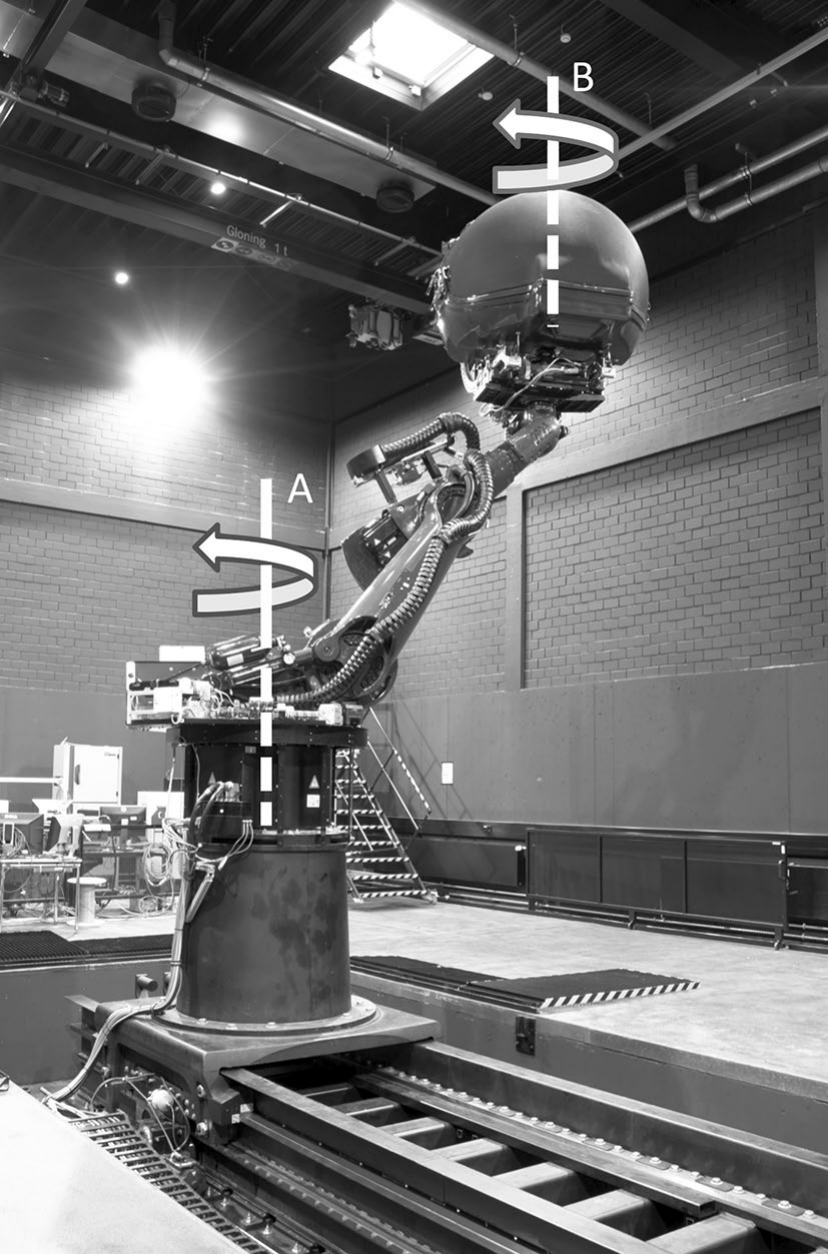
CyberMotion simulator in the configuration for the circular trajectories. During the trial, the cabin was rotated using the main *centrifuge axis* (**a**); Only after the trial, the cabin was reoriented using the cabin *yaw axis* (**b**)

The study was performed using the MPI CyberMotion simulator (Nieuwenhuizen and Bülthoff 2013), which consists of a robotic arm on top of an earth vertical yaw axis (Fig. 3). The participant was seated inside the enclosed simulator cabin at the end of the arm and secured with a five-point seat belt. During the experiment, the cabin was moved along a circular path in the horizontal plane, with the participant always erect and facing the direction of motion, equal to the example shown in Fig. 2a, b. The yaw velocity profile was a raised cosine bell with a maximum of 60°/s and a period of 12 s. With a radius of 1.93 m, this resulted in a maximum tangential acceleration of 0.53 m/s^2^ and a centripetal acceleration of 2.11 m/s^2^. Total angular displacement was 360°, and the motion direction was randomly chosen clock- or counterclockwise.

Audio communication between experimenter and participant was possible at all times through a headset. An infrared video camera inside the cabin allowed further monitoring of the participant. Whenever the simulator was moving, auditory white noise was provided to mask environmental sounds. Participants were blindfolded and wore a neck brace to help keeping the head upright and minimize any head movements with respect to the trunk. They were unaware of the exact motion capabilities of the simulator, and on entering the simulator, the configuration of the arm differed from the one used during the experiment. Furthermore, participants had no knowledge about the motion profile, other than it being a motion “along a curved path in the horizontal plane.” While in these trials they were always facing the direction of motion, participants were told that their heading could be manipulated. At the beginning of each trial, the simulator cabin was moved slowly into the start position. After a 5-s pause, the motion started, with the start and stop indicated by an auditory signal. After the trajectory, a rapid reorientation motion followed, consisting of 2–4 semi-random rotations to the left and/or right. Note that this rotation was around the body yaw axis (i.e., on-center) and not around the centrifuge axis (Fig. 3). As confirmed by a pilot study, this reorientation prevented tracking of one’s yaw orientation in space and ensured that participants were not aware of the heading at the start of the next trial. They were instructed to start the next trial only when all motion after-effects had vanished.

Perceived yaw rotation was assessed in a block of four trials, using a continuous pointing task. Here, participants were instructed to point a pointing device toward an imaginary earth-fixed reference. That is, they were asked to imagine a distant, earth-fixed object at the horizon at the start of the trial and continuously point toward this object during the trial. In this way, they attempted to keep the pointer stationary in an earth reference frame, like the needle of a compass. The pointer allowed for unlimited rotation in the horizontal plane and was fixed to the participant’s chair in the simulator cabin. Its angular displacement was measured using an absolute magnetic encoder (R9000, IFM electronics) with a resolution of 0.1° and was taken as a measure for perceived yaw rotation. This was compared to both the physical yaw rotation and the model prediction using Wilcoxon signed rank tests. In a second block of four trials, the perceived motion path was reported through a drawing, made right after the end of the curved trajectory. Here, participants also indicated their perceived heading at the start, middle, and end of the motion. In these trials, the rapid reorientation was postponed for 30 s, leaving sufficient time to perform the task. The drawings were used to obtain a general impression of the perceived motion path and were not used for hypothesis testing. Both blocks of trials were presented in random order. The first trial of each block was regarded as training and was excluded from further analysis.

### Results

**Fig. 4:**
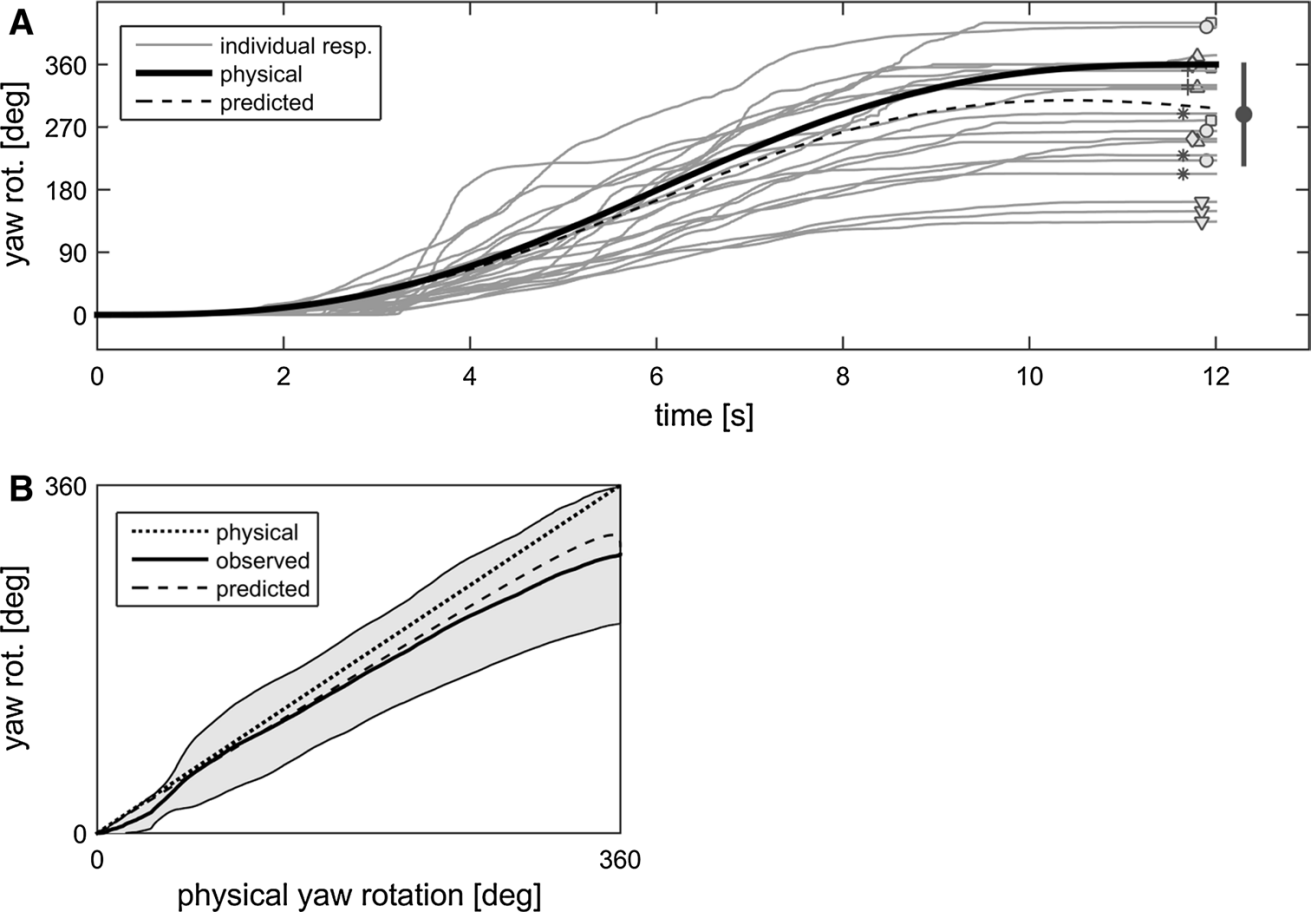
Individual pointing responses for all participants (labeled with *different symbols*) and repetitions (**a**) together with the physical and predicted perceived rotation. The group mean and SD in the final angle are indicated on the *right*. **b** The average response (*black solid line*), SD (*shaded areas*), and the predicted rotation (*dashed line*) versus the physical rotation. The *dotted line* is the 1:1 line

The pointing responses (Fig. 4a) showed a gradual increase in perceived yaw rotation over time. Looking at the final angle, participants were reasonably consistent in their performance, with an average intra-individual variability of 53°. The final angle was underestimated, with an average of 288° (SD = 71°) over all participants. This was significantly different from the physical displacement (360°, *p* = 0.02, *z* = −2.37), but not from the model prediction (289°, *p* = 0.74, *z* = 0.34). Figure 4b shows how the observed responses relate to the physical and predicted perceived yaw rotation, respectively, over the course of the trial. Regression analyses performed on the individual data showed that the observed responses were related linearly with both the physical motion and the predicted percept (*r* ≥ 0.97 in all cases). This indicated that participants followed the motion dynamics correctly. Importantly, the regression line of the observed response versus the physical rotation had a slope significantly lower than 1 (mean slope 0.83, SD = 0.23, *p* = 0.03, *z* = −2.20), which is in line with the underestimation of the total displacement shown above. The regression line of the observed response versus the predicted percept, on the other hand, had a slope not significantly different from 1 (mean = 0.94, SD = 0.24), indicating that the responses were in line with the model predictions.

**Fig. 5:**
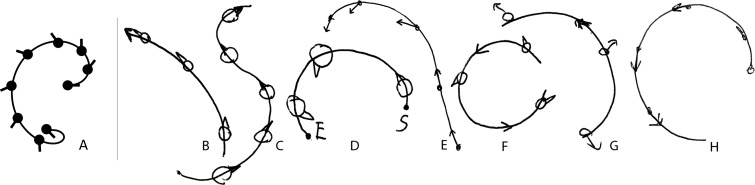
Model prediction (**a**) and examples of individual drawings of the perceived path (**b**–**h**)

For the perceived travelled path participants generally drew similar shapes over the repeated trials, but shapes varied between participants (Fig. 5). Three categories could be discerned: An arc (i.e., constant radius curve) was observed in 57 % (Fig. 5b, d, f–h), a spiral (i.e., varying radius, Fig. 5e) in 24 %, and an S-shape (i.e., change in rotation direction) in 19 % (Fig. 5c) of the observations (21 in total).

Interestingly, the drawings were not always consistent with the pointing data. Whereas the perceived yaw rotation followed a monotonically increasing pattern (Fig. 4), the drawings of the latter two categories would require a non-monotonous increase (spiral) or a change in sign (S-shape). A few participants also noticed the inconsistency themselves, either in shape of the drawn trajectory, or in the amplitude of the rotation. That is, the angular displacement in the pointing task was generally larger than the rotation amplitude as inferred from the drawing. The latter measure was obtained by comparing the indicated head orientation in space at the start and end of the drawn path. Lastly, the perceived head orientation with respect to the path, as indicated on the drawings, also varied considerably between participants, covering the whole range from facing outward to inward and being aligned with the motion direction. In the majority of the responses (67 %), the perceived heading remained constant during the whole trajectory (e.g., Fig. 5b, c, f–h).

### Discussion

On average, the yaw rotation in space was underestimated, which is in line with the model predictions. The large inter-individual variability in the data was also observed by Ivanenko et al. (1997a, b), using a similar tracking task during a 180° turn trajectory. In general, large differences between individuals are common in motion perception studies (e.g., Benson et al. 1989; MacNeilage et al. 2010b). In the specific case of yaw rotation perception during prolonged motions, this is typically explained in terms of individual differences in the so-called velocity storage time constant (Raphan et al. 1979). Velocity storage refers to the central integration of rotational signals and decreases the typical decay in the SCC signals during prolonged rotation. The time constant of this integration has been shown to differ largely between individuals (Bos et al. 2002; Bertolini et al. 2011), leading to differences in the decay rate of perceived rotation.

Regarding the travelled path, the combined translational and rotational stimuli resulted in a whole range of different percepts, which were qualitatively inconsistent with the perceived yaw rotation as obtained in the tracking task in at least two of the seven participants. This raises the question whether such an offline drawing task provides a valid measure for travelled path shape. During the debriefing, many participants responded they found the drawing task difficult, which suggests that it was by no means straightforward to combine all motion cues into one overall motion percept, even for such a familiar motion (e.g., car driving). A factor possibly contributing to this is the lack of prior knowledge on both motion path and heading. The most frequent observed shape, however, was an arc, in accordance with the results of Ivanenko et al. (1997b). The drawings, although coarse, provided no strong evidence for the heading to change over the course of the trajectory. Indeed, some participants felt being faced outwards during the curve (as the model predicts), but others felt being aligned, or being faced inwards. Therefore, in Experiment 2 we focused on testing this one specific and distinct prediction: namely that a perceived heading bias would occur as the curve proceeds.

## Experiment 2: heading perception

The goal of the second experiment was to investigate heading perception during circular trajectories, both in darkness and with visual stimulation. Using an adaptive psychophysical procedure, we determined the physical heading that was perceived as straight ahead (i.e., facing the motion direction) to identify possible heading bias. Although not directly the focus of this paper, this method also allowed us to measure heading sensitivity in curves.

### Methods

**Fig. 6:**
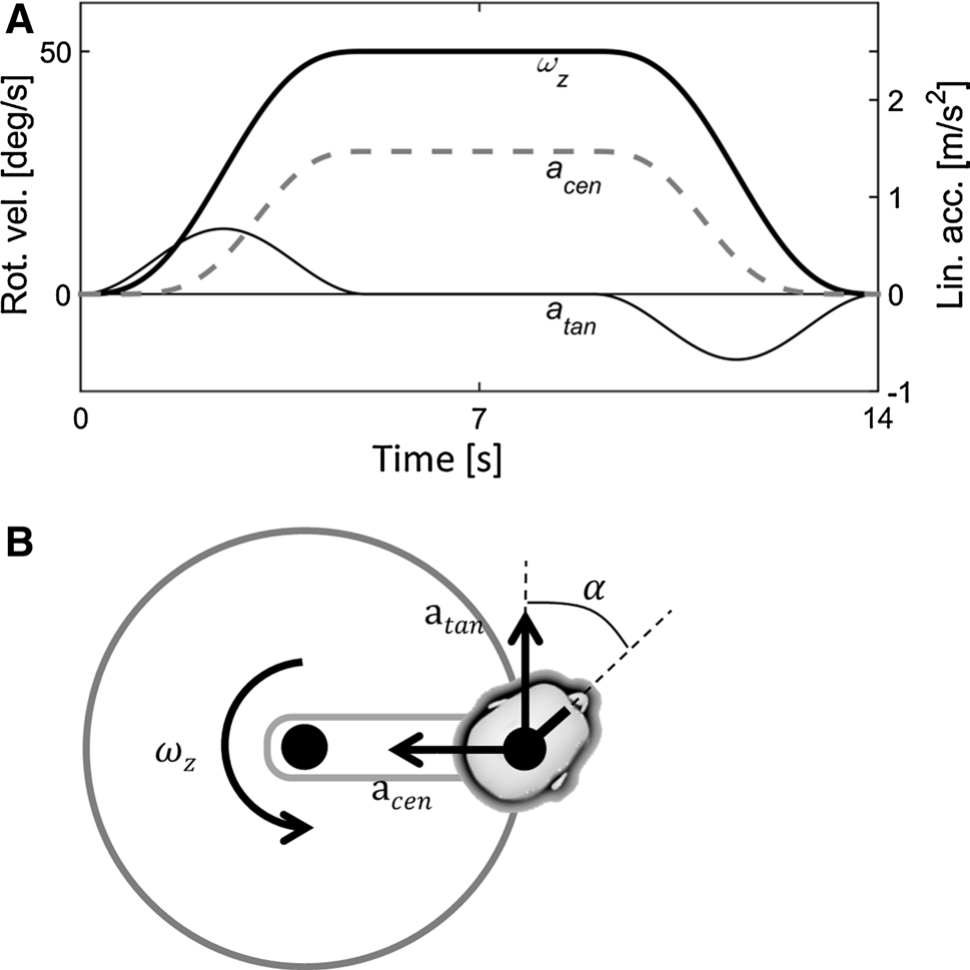
Stimulus profile of Experiment 2 (**a**), where $\omega_{z}$ = yaw velocity, $a_{\tan }$ = tangential acceleration, and $a_{\text{cen}}$ = centripetal acceleration. How these linear accelerations were acting on the body was determined by the participants heading ($\alpha$), which was varied between trials (**b**)

Equipment and procedures were largely similar to those of Experiment 1. Six healthy volunteers (four males and two females, aged between 22 and 27, and all different from those of Experiment 1) participated in Experiment 2. Participants were repeatedly exposed to circular trajectories, but now the heading was systematically varied between trials. Instead of always facing the motion direction (as in Experiment 1), they were oriented toward the center of the circle, or away from it (see Fig. 6). Throughout the trial, this orientation with respect to the motion path was kept constant and no head-on-body movements were present. The rotational velocity followed a trapezoid-like profile. At the start of the trial, the velocity was increased following a half raised cosine profile to 50°/s, with a peak acceleration of 20°/s^2^. The final velocity was maintained for 4 s, after which deceleration started. The deceleration mirrored the acceleration phase. The maximum tangential acceleration, occurring during acceleration/deceleration, was 0.67 m/s^2^, and the maximum centripetal acceleration, occurring during constant velocity, was 1.47 m/s^2^. The total duration of the motion was 14 s, and the total yaw rotation equaled 450°.

The experiment consisted of two experimental sessions, carried out on two separate days. In the first session, participants were blindfolded to assess heading perception in darkness (DARK). In the second session (LIGHT), the motion was combined with a congruent visual stimulus (i.e., a visual scene rotating with equal intensity as the motion stimulus and opposite direction). Visual stimuli (Virtools, 3DVIA) were projected on the white, curved inner surface of the cabin (FoV 160° × 90°), and consisted of a horizontal ground plane formed by random dots. Therefore, the stimulus provided information on one’s orientation with respect to gravity and one’s movement velocity. A new dot plane was constructed at the beginning of every trial, to prevent the recognition of one’s position on the plane. Fog was provided (density increased with exponential squared distance) to reduce the visibility of distant dots. To ensure natural viewing behavior, no fixation cross was provided (Li et al. 2006).

Prior to the experiment, participants were told that the motion would be a circular motion of undefined length and that their heading would be manipulated by changing their physical yaw orientation with respect to the path (denoted here as yaw offset). They were explained that this manipulation would change the direction of the force they perceived during the circular motion. In a training session, they were exposed to several trials with their yaw offset ranging from 90° inward (i.e., facing toward the center of the circle) to 90° outward (facing away from the center). The experimenter ensured that these extreme conditions were indeed perceived correctly as facing in- or outward. No feedback was provided on the intermediate training angles.

Halfway through each trial an auditory signal was played (denoted as “target beep”). The participant had then to answer the following question: “Was your nose left or right from the motion path at the moment of the target beep?” The participant was instructed to respond as fast as possible by means of a button press. When no answer was recorded before the end of the circular trajectory, the trial was repeated. The trajectory was followed by the rapid reorientation (see Experiment 1), and the next trial was self-initiated when the participant felt stationary again.

**Fig. 7:**
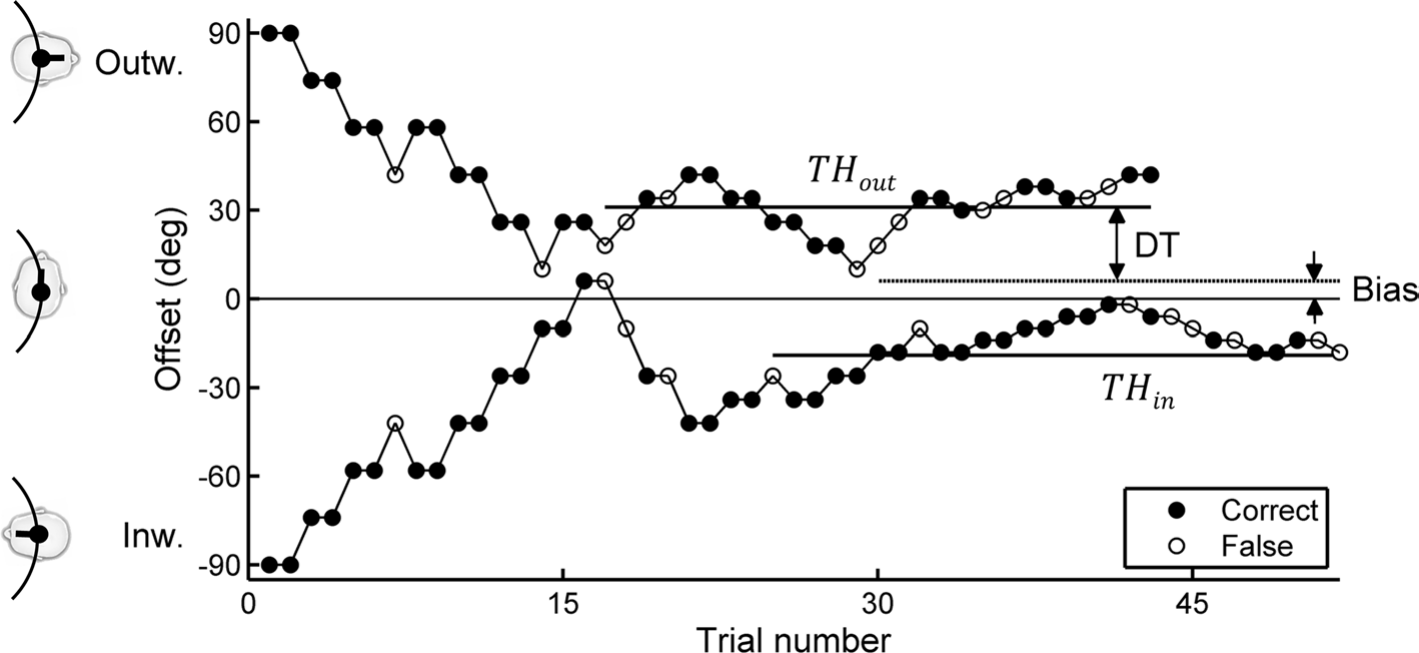
Example of the staircase results for one participant, starting at a yaw offset from 90° in- or outward, and converging to the threshold level TH_in_ and TH_out_, respectively. From these, the bias and differential threshold (DT) were calculated

The yaw offset for the next trial was determined by a psychophysical adaptive procedure using two randomly interleaved staircases (Levitt 1971). The staircases started at a yaw offset of 90° in- and outward. Following a 2-down-1-up rule, the yaw offset was decreased after two subsequent correct answers and increased after one false answer. The initial step size for these adjustments was 16° and was halved after every four reversals (i.e., when the adjustment changed from a decrease to an increase or vice versa). The staircase was terminated after 12 reversals. A typical example of the staircases is shown in Fig. 7. On average, participants needed 49 trials to complete one staircase (SD = 5). Including repositioning, one trial lasted about 45 s, and participants generally needed about 90 min to complete the training and all experimental trials. Breaks were provided every 30 min, or more often when requested.

The adaptive procedure with the 2-down-1-up rule allowed us to determine the physical yaw orientation that the participant consistently identified as in- or outwards in 70.7 % of the cases. These threshold levels were calculated as the average heading over the last eight reversals and are denoted as TH_in_ and TH_out_, respectively (Fig. 7). Heading bias was then defined as the average of TH_in_ and TH_out_ and reflects the physical orientation that is *perceived* as facing the motion direction. The differential threshold (DT) was used as a measure of sensitivity and was defined as the difference between the heading bias and the thresholds TH_in_ and TH_out_, respectively. It reflects the difference required to correctly perceive the heading as being different from the perceived aligned condition in 70.7 % of the trials. Note that the DT is inversely related to precision: The smaller the DT, the more precisely a participant can indicate his/her heading. Because of the limited number of participants, nonparametric statistics (Wilcoxon signed rank test) were used to test for differences between conditions and between observations and model predictions.

### Results

**Fig. 8:**
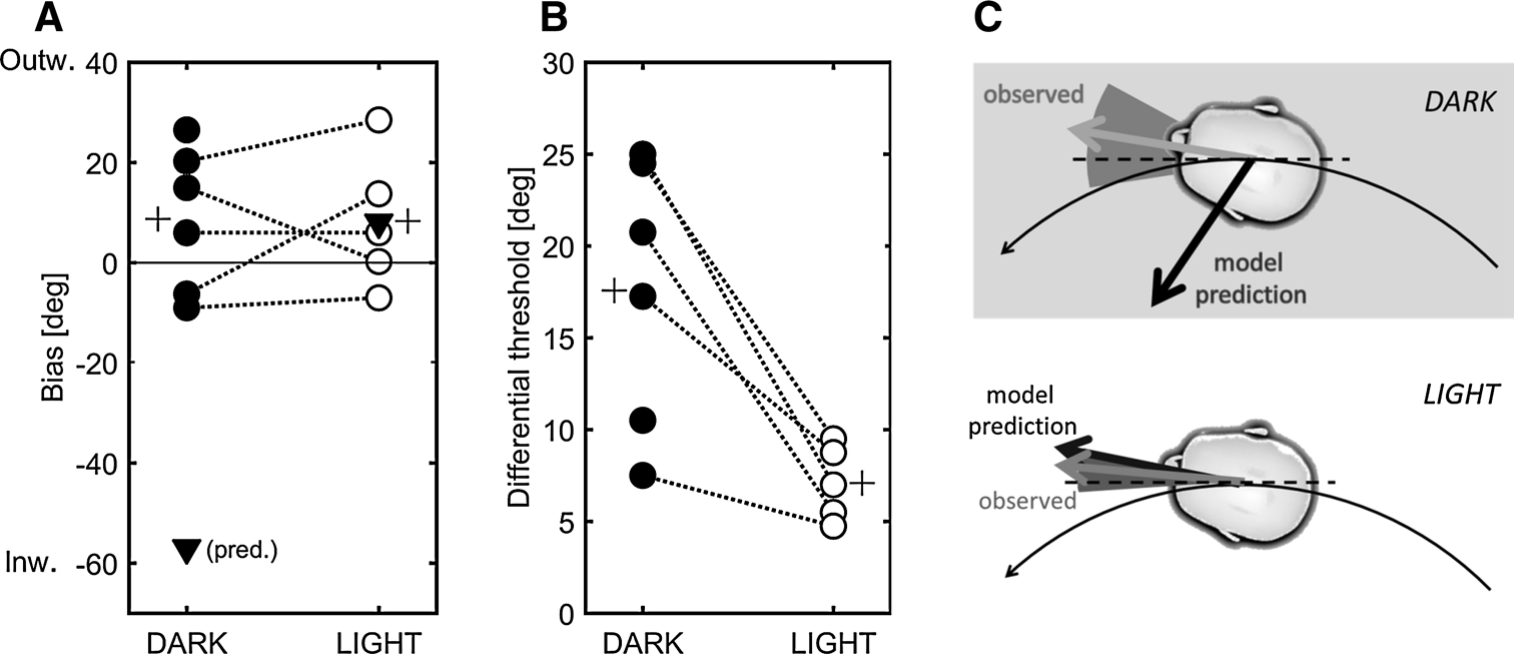
Individual results for heading bias (**a**) and differential threshold (**b**). The group average is indicated by the *plus sign*, and the model-predicted bias is shown by the *triangle*. Group results are summarized in **c**. The *arrows* indicate the average bias, i.e., the physical orientation required for the perceptual straight ahead. The *shaded triangles* indicate the average differential threshold

 Figure 8 summarizes the results of Experiment 2, including the model predictions for the expected bias. These model predictions were obtained by calculating the motion cues acting on the body for the whole heading range that was tested, and using these as input to the perception model. The model predicts that, in order to perceive the heading as “straight ahead”, a physical orientation of 57° *inward* would be required (triangle in Fig. 8a). In the LIGHT condition, the perceptual straight ahead would require a physical orientation of 8° *outward*. For technical reasons, data for the LIGHT condition could not be obtained in one of the six participants.

The results show that the measured bias differed largely between individuals (Fig. 8a). In the DARK condition, the mean bias was +8.8° (SD = 14.4°), meaning that, on average, the orientation for the perceptual straight ahead is slightly outward. Although not different from the physical straight ahead (*p* = 0.25, *z* = 1.15), these results are clearly different from the 57° inward orientation as predicted by the perception model (*p* = 0.028, *z* = 2.20). The bias was not affected by the visual stimulus: In LIGHT, mean bias was +8.3° (outward, SD = 13.6°). This was different neither from the actual straight ahead (*p* = 0.23, *z* = 1.21), nor from the model prediction of 8° outward (*p* = 0.89, *z* = −0.14).

The visual stimulus did, on the other hand, cause a decrease in the DT for perceiving the straight ahead. The average DT equaled 17.5° in the DARK (SD = 7.3°) and significantly decreased (*p* = 0.043, *z* = −2.02) to 7.1° (SD = 2.3°) in the LIGHT condition (Fig. 8b).

The average response time over all participants was 1.14 s (SD = 0.46 s) in DARK and 0.96 s (SD = 0.26 s) in LIGHT. This decrease was not significant. The average intra-individual variability (SD) was 0.76 and 0.66 s, respectively. With the target beep being halfway the 4 s velocity plateau, these response times indicate that participants were generally able to respond within the time where the motion stimulus was constant.

### Discussion

In Experiment 2, we found no evidence for the consistent heading bias predicted by the model for circular motion in darkness. Both in darkness and in light, the heading bias was not significantly different from 0°. To our knowledge, there are no data available for comparison on heading bias for curved trajectories in darkness. Data on pure visual heading perception in curves (i.e., observer stationary) are in line with our results (Stone and Perrone 1997; Li et al. 2006, 2009). Stone and Perrone used a psychophysical approach similar to ours and found biases of about 3°–6°. Similar values were also reported by Li et al. (2006, 2009), who used an interactive task where participants had to actively align the camera view direction with the tangent of the curve. Over all participants, the bias they observed was not significantly different from 0°.

Similar to Experiment 1, the inter-individual variability in the bias was considerable. Although large inter-individual variability is a common finding in human perception studies, it exceeds what is generally observed for linear heading perception (i.e., on straight paths). For example, Cuturi and MacNeilage (2013) measured bias and sensitivity for linear heading perception as a function of motion direction and reported an inter-individual variability (SD) of about 6° for heading bias in darkness, independent of movement direction. In the current study, this was 14°. In addition to inter-individual differences in the central processing of rotational signals, mentioned in Experiment 1, another factor that likely contributes to this variability is the increased complexity of the stimulus (curved vs. linear motion), and, relatedly, the participant’s understanding of the motion dynamics. As emerged from a pilot study, knowledge about the specific motion profile appeared important for the task. When the motion dynamics were not explained on forehand, many participants became very insecure about their answers and inter-individual differences were even larger. A proper understanding of the relationship between the experienced yaw velocity and the perceived forces helped in reducing the variability between participants. It indicates that understanding the motion dynamics helped in focusing on the task-relevant information.

Besides heading biases, Experiment 2 further allowed to measure heading sensitivity, or precision. The visual stimulus made the heading estimate more precise, as indicated by the lower DT in the LIGHT condition. This is in agreement with results on linear heading perception, where the precision is also found to increase when visual information is also present (e.g., Telford et al. 1995; Cuturi and MacNeilage 2013; De Winkel et al. 2010; Butler et al. 2010; Butler et al. 2015). We have to note that it is possible that the amount of experience with the task might have contributed to the effect. Because the DARK condition was most important in evaluating the model predictions, we choose to have a fixed order of conditions, so that the amount of training was equal for all participants. A training effect can therefore not be ruled out.

Our results indicate that the sensitivity to identify the straight ahead during curved motions is much lower than what is generally found for straight motions. In many studies, the sensitivity is characterized by the standard deviation *σ* of the cumulative normal distribution that is assumed to underlie the responses (Cuturi and MacNeilage 2013). It reflects the DT at the 84 % correct level. An estimate for *σ* can be obtained from our data by dividing the DT at the 70.7 % correct level (that followed directly from the 2-down-1-up staircase) by a factor of 0.54 (Levitt 1971). For our participants, this would result in a mean *σ* of 32° in the DARK condition. Since the main cue informing about heading in our motion profile was the lateral centripetal acceleration, our data can best be compared with heading sensitivity to linear *lateral* motion. For a lateral acceleration stimulus, Cuturi and MacNeilage (2013) report a *σ* of about 8°, which is about four times lower than what we find for circular paths. Whereas the magnitude of the acceleration stimulus of Cuturi and MacNeilage (1.13 m/s^2^) was comparable to ours (1.47 m/s^2^), the duration of their stimulus was much shorter (1 s). Although we cannot rule out any effect of stimulus duration on the sensitivity, we anticipate that such an effect would be limited, because both stimuli were well above the perception threshold. More likely, the fact that the linear acceleration stimulus was presented with concurrent rotation explains the lower heading sensitivity we observed. It is a common finding that the presence of motion cues that are not relevant for the task deteriorates the sensitivity to the task-relevant motion cues (Zaichik et al. 1999; MacNeilage et al. 2010b; Pretto et al. 2014). In contrast, added motion cues tend to increase sensitivity when they are relevant for the task, as, for instance, shown by the increased sensitivity with added congruent visual information (e.g., Telford et al. 1995 Cuturi and MacNeilage 2013; De Winkel et al. 2010; Butler et al. 2010, 2015).

## General discussion

By measuring different aspects of motion perception during circular trajectories, both in darkness and with congruent visual motion information, we tried to identify to which extent the perceived motion could be understood by the well-known perceptual mechanisms within the CNS that are formalized in a mathematical model for human motion perception. Different models have been developed over the years (Mayne 1974; Merfeld et al. 1993; Bos and Bles 2002; Zupan et al. 2002; Laurens and Droulez 2007; Newman 2009), but they are similar in the sense that they all take the main perceptual mechanisms into account: sensory dynamics, the tilt-translation resolution, and the somatogravic illusion. Here, we used the model based on Merfeld and colleagues, because it included estimates of the perceived translation through the environment, and the integration with visual signals.

The perception model provided separate estimates of rotation and translation, and both components were then summed to provide the overall predicted travelled path through the environment, and the heading. For a circular motion in darkness, the model predicted that perceived rotation would be fairly accurate (only slightly underestimated), but that the perceived motion path and the perceived heading (reflecting one’s orientation with respect to the path) would be distorted. The predicted path deviates from being a circle and moving with the head aligned to the motion would be perceived as facing outward. Experiment 1 was set up to measure perceived rotation and travelled path, whereas Experiment 2 investigated perceived heading. The main result of this paper is that rotation perception was in line with the model predictions but that heading perception was not: It was more veridical than the model predicted. As such, the rotation and translation components seem to be processed in a more integrated way than it is assumed by the model. This would be in line with earlier conclusions of Ivanenko et al. (1997a, b), who argued that the overall perceived motion through the environment does not seem to follow from the simple addition of the perceived translational and rotational components. Below we will elaborate on possible explanations for these findings and their implications.

The first question that arises is whether the predicted heading bias was dependent on the choice of model parameter values. Would other parameters lead to qualitatively different predictions? To gain insight into this issue, multiple simulations were performed using a range of different parameter sets (see Appendix 2). Decreasing or increasing all parameters up to a factor five did not qualitatively change the predicted bias: In all cases, the model predicted a perceived *outward* orientation for a physical orientation that was aligned with the direction of motion. A predicted perceived inward orientation, which would be more in accordance with the individual responses found in Experiment 2, was never observed. This indicated that the model in itself was not able to capture the observed responses, and therefore no fitting of the model parameters to the original responses was performed. To assess the generalizability of the predictions, simulations were also performed using a more generic perception model, as the one proposed by Holly et al. (2008). Their so-called standard model accounted for the main characteristics of many current perception models, but used a more generic structure. These simulations corroborated our earlier results. From this, we concluded that it is only possible to reduce the predicted bias to zero by eliminating all dynamics that are accounted for in the model, that is, when eliminating the somatogravic effect plus the decay in the perceived translation and rotation. We consider this a highly unlikely solution. First of all, our results of Experiment 1 support the underestimation of the perceived rotation over time, in line with other studies on eccentric rotation (Mittelstaedt and Mittelstaedt 1996). Second, although perceived tilt was not directly measured in this study, most participants reported tilt when asked to describe their motion percept during the study debriefing. The applied motion stimulus provided a 9° tilt of the gravito-inertial acceleration (GIA), which is well above the tilt perception threshold. For physical whole-body tilt, thresholds of 1.5° or lower have been reported (Valko et al. 2012), whereas for tilt induced by centrifugation, as was done in the current study, the threshold is around 3°–4° (Janssen et al. 2011). Indeed, Graybiel and Clark (1965) showed that a GIA tilt of 10° elicited noticeable tilt responses in their participants. Given these considerations, we believe that tilt was perceived with our stimulus, which would have affected the perceived travelled path and therefore the heading. In future experiments, tilt measurements should therefore be considered.

If the perceptual dynamics cannot be discarded, as argued in the previous paragraph, it follows that we cannot explain the observed veridical heading percept with the current perception model. Are there factors that could have prevented the heading bias to occur, that are currently not accounted for by the model? One possibility is that the perceived heading at the start of the trial governed the percept during the rest of the motion. The importance of the initial percept is nicely shown in a study of Bertin and Berthoz (2004), who investigated the perception of the travelled path under the influence of ambiguous optic flow patterns that contained both linear and rotational components. In that study, a short inertial motion was presented only very briefly at the start of the visual stimulus, and the authors showed that this shaped the subsequent visual motion percept. That is, brief translational inertial motion enhanced perception of the translational part of the optic flow, whereas rotational inertial motion enhanced the perception of the rotational part of the optic flow. In our study, the model predicted that the aligned heading would quickly be perceived as biased when the motion progressed. However, if the initial part of the motion is able to shape the perception of the subsequent part, the emergence of the bias would be prevented. This would also be in line with our observation that in the drawing task of Experiment 1, most participants perceived a curve with a stable heading over time.

Another factor that gained interest over the last years is the possibility that the overall motion percept relies (at least partly) on familiar, or expected stimulus patterns. Firstly, knowledge of the apparatus’ motion capabilities is known to affect the reported percept (Wertheim et al. 2001). Secondly, motion history and expectation is likely to play a role. Using a Bayesian framework, Prsa et al. (2015) showed that the magnitude of perceived yaw rotation was drawn toward the running average of the preceding trials when all trials were randomly chosen (motion history). On the other hand, when the amplitude of consecutive trials followed a specific pattern, participants were able to take that into account. Related to this, Rader et al. (2009) showed that participants seem to take the expected motion dynamics into account. They measured motion perception during roll tilt on a swing device where the rotation radius could be varied. Although not always veridical, participants’ reports showed internal consistency, that is, their reports of perceived tilt, translation, and the estimated swing radius were related according to swing geometry.

Current models generally do not account for factors such as expectation or knowledge about the lawful relationships between rotational and translational variables (i.e., motion dynamics), although attempts in this direction have been made (Holly et al. 2008; Rader et al. 2009; Prsa et al. 2015). Holly et al. (2008) tried to explain observed differences in roll tilt perception during forward- and backward-facing centrifuge runs by incorporating concepts of familiarity. This included the geometry of circular motions ($a = \omega \times v$), that is, the fact that roll tilt of the GIA is a characteristic of a motion through a curve at a particular radius and thus does not necessarily indicate body tilt. Together with the assumption that humans were more familiar to forward motion than to backward motion, this was sufficient to explain experimental results that could not be explained by the current models without such components.

Our finding that the model-predicted heading bias was not experimentally observed could also hint at such a mechanism. It suggests that the overall motion percept is not just the sum of the perceived linear and angular parts, in line with the previous results of Ivanenko et al. (1997b). Possibly, the motion cues are processed in congruency, taking into account one’s expectations regarding the subjected motion and the expected relationship between its components. Experimentally manipulating this expectation is therefore needed in future research.

In conclusion, the results of this study show that motion perception in circular trajectories is more veridical than what would be predicted by current perception models. This suggests that the overall perceived motion is not just the sum of independently processed rotational and linear cues. Likely, higher-order processes, like the expected relationships between different components, are also considered within the CNS.

## Figures and Tables

**Fig. 9:**
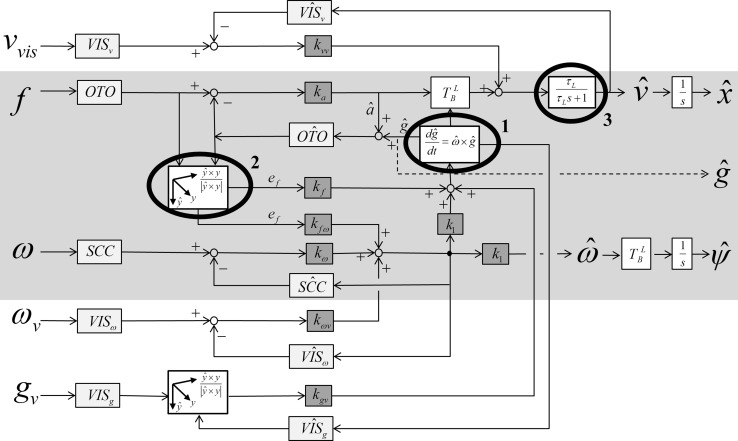
Schematic overview of the motion perception model based on Merfeld et al. (1993) and Newman (2009). The *vestibular part* is shown in the middle, on the *gray* background. *Sensors* are indicated in *light gray*, feedback gains in *dark gray*, and other mathematical operations in *white*

**Fig. 10:**
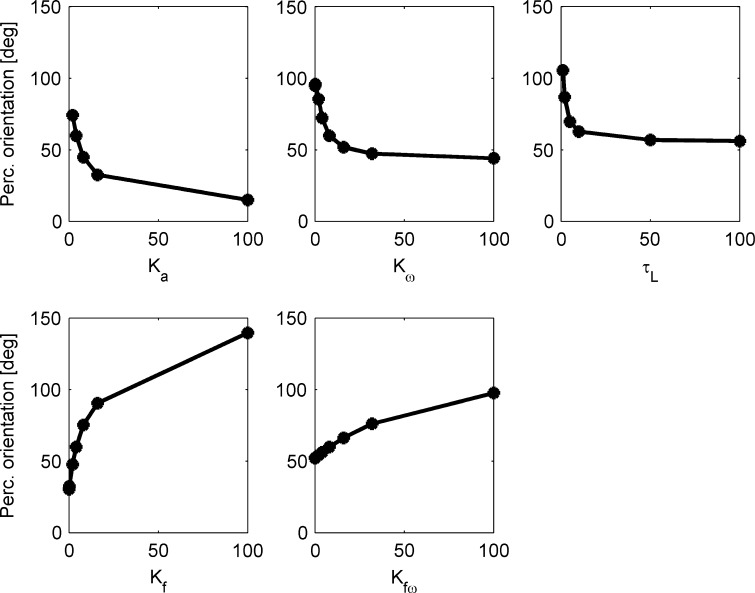
Predicted yaw orientation w.r.t. the motion path during a circular motion in darkness (*positive values* indicate a perceived outward orientation). Simulations were performed by changing one parameter value at the time, and keeping all others at their default value and using the motion profile of Experiment 2 with the heading straight ahead

## Appendix 1: Self-motion perception model

Predictions for the perceived motions were obtained running simulations using a mathematical model for spatial orientation that included both visual and vestibular processing paths (Newman 2009). Its vestibular core is formed by the well-known model of Merfeld et al. (1993), which has been extended recently to include visual–vestibular interaction and outputs for linear and angular displacement. Below the general layout of the model is summarized; for a detailed explanation of the model, we refer to the original manuscripts (Merfeld et al. 1993; Newman 2009; Newman et al. 2012).

The model is a co-called observer model, of which the primary assumption is that the CNS incorporates information about the sensor dynamics and physical laws to form an internal model that predicts the sensory signals that results from a certain movement. This expected sensory signal is then compared to the actual sensory signal, and the error between the two is then used to drive the central perceptual estimates toward more accurate values.

The model is shown in Fig. 9, with the vestibular core in gray. Inputs to the model are inertial angular velocity (*ω*), gravito-inertial acceleration (*f*), and, if appropriate, visual linear velocity (*v*_vis_), visual angular velocity (*ω*_vis_), and visual orientation with respect to gravity (*g*_vis_). The signals are first fed through the different visual and vestibular sensors. The SCC dynamics are modeled by a second-order high-pass filter using a short time constant of 5 s to represent the cupula-endolymph dynamics and a long 80-s neural adaptation constant to represent response decay to constant acceleratory stimuli (Merfeld et al. 2005b; Newman 2009). For the otoliths (OTO) and the visual sensors (VIS_*ω*_, VIS_*v*_, VIS_*g*_), an identity matrix was used.^2^ As shown in Fig. 9, the output of each sensor is then compared to the output of the internal model of this sensor (indicated by a hat sign), which is assumed to have similar dynamics. The difference is weighted by a factor *K* and fed back into the internal model.

These weighted error signals form the basis of the internal estimates on which the percept is based. For the linear acceleration estimate $\hat{a}$, this difference is weighted by gain *K*_*a*_ and with *K*_*ω*_ for the rotational velocity estimate $\hat{\omega }$. An additional gain *K*_1_ (*K*_1_ = (*K*_*ω*_ + 1)/*K*_*ω*_) was added in the rotational velocity feedback loop to ensure that the overall loop gain equaled 1. The estimate for gravity $\hat{g}$ is obtained by integrating $\hat{\omega }$ over time (Fig. 9, operation 1). The tendency for $\hat{g}$ to align with the net otolith signal (i.e., the somatogravic illusion) is accounted for by operation 2. Here, a rotation error signal (*e*_*f*_) is calculated that would align the actual and the expected otolith signal. This rotation error affects $\hat{g}$ directly through *K*_*f*_, and indirectly via *K*_*fω*_, through its effect on $\hat{\omega }$. The linear acceleration estimate is transformed into an external frame of reference (*T*_*B*_^*L*^) and then fed through a leaky integrator to obtain linear velocity ($\hat{v}$, operation 3), and after one more integration, displacement ($\hat{x}$). Yaw angular displacement in space ($\hat{\psi }$) is derived from integrating the earth vertical component of $\hat{\omega }$. Visual information is processed in a similar way and affects $\hat{\omega }$ through the gain *K*_*ωv*_, $\hat{v}$ through the gain *K*_*vv*_, and $\hat{g}$ through the gain *K*_*gv*_. The model was implemented using MATLAB and MATLAB Simulink R2013a. Parameter values used for the simulations presented in this paper were similar to those of Newman (2009): *K*_*a*_ = 4^3^; *K*_*ω*_ = 4; *K*_*f*_ = 8; *K*_*fω*_ = 8; *K*_*ωv*_ = 10; *K*_*vv*_ = 0.75; *K*_*gv*_ = 10; *τ*_*L*_ = 16.67 s for horizontal motions and 1 s for vertical.

## Appendix 2: Effect of parameter values on heading bias

As is shown in Fig. 2, the model predicted that a heading bias would develop during a circular motion in darkness. The impact of parameter values on the heading bias was assessed by systematically changing the values for *K*_*a*_, *K*_*ω*_, *K*_*f*_, *K*_*fω*_, and *τ*_*L*_ (note that the value of *τ*_*L*_ is different for the horizontal and vertical dimensions. Here, only the horizontal dimension was varied). Figure 10 shows the effect of varying one parameter at the time, while keeping the others fixed at their default values. Generally speaking, the perceived motion becomes more veridical by increasing the values for *K*_*ω*_, *K*_*a*_, and *τ*_*L*_ and decreasing the values for *K*_*f*_ and *K*_*fω*_. Note that the latter two determine the perceived tilt and thus have a large effect on the bias. When these two parameters are both set to 0 (i.e., no tilt), and the others remain at their default value only a small outward bias of 10° is predicted. Thus, the magnitude of the predicted bias can be reduced by increasing the values of *K*_*ω*_, *K*_*a*_, and *τ*_*L*_ and decreasing the values for *K*_*f*_ and *K*_*fω*_. Simulations were run by using all possible parameter combinations using multiplication factors between 0.2 and 5. The predicted bias ranged between 7° and 177° outward, whereas no inward biases were observed. This indicates that, in order to perceive one’s heading as aligned with the motion direction, the physical orientation has to be inward in all cases tested.

## Footnotes

1 The word curve is used here to indicate that the shape of the perceived path is non-straight, but does not represent the actual circle.
